# Cancer Care Terminology in African Languages

**DOI:** 10.1001/jamanetworkopen.2024.31128

**Published:** 2024-08-30

**Authors:** Hannah Simba, Miriam Mutebi, Moses Galukande, Yahya Mahamat-Saleh, Elom Aglago, Adamu Addissie, Lidya Genene Abebe, Justina Onwuka, Grace Akinyi Odongo, Felix M. Onyije, Bernadette Chimera, Melitah Motlhale, Neimar de Paula Silva, Desiree Malope, Clement T. Narh, Elizabeth F. Msoka, Joachim Schüz, Efua Prah, Valerie McCormack

**Affiliations:** 1 Environment and Lifestyle Epidemiology Branch, International Agency for Research on Cancer (IARC), World Health Organization (WHO), Lyon, France; 2 Department of Global Health, Stellenbosch University, Cape Town, South Africa; 3 Department of Surgery, Aga Khan University, Nairobi, Kenya; 4 Department of Surgery, College of Health Sciences, Makerere University, Kampala, Uganda; 5 Nutrition and Metabolism Branch, IARC, WHO, Lyon, France; 6 Department of Epidemiology and Biostatistics, School of Public Health, Imperial College London, London, United Kingdom; 7 Department of Preventive Medicine, School of Public Health, College of Health Sciences, Addis Ababa University, Addis Ababa, Ethiopia; 8 Genetics Branch, IARC, WHO, Lyon, France; 9 Epigenomics and Mechanisms Branch, IARC, WHO, Lyon, France; 10 National Cancer Registry, National Health Laboratory Service, Johannesburg, South Africa; 11 Cancer Surveillance Branch, IARC, WHO, Lyon, France; 12 Strengthening Oncology Services Research Unit, Faculty of Health Sciences, University of the Witwatersrand, Johannesburg, South Africa; 13 Department of Epidemiology & Biostatistics, Fred N. Binka School of Public Health, University of Health and Allied Sciences, Hohoe, Ghana; 14 Department of Community Health, Institute of Public Health, Kilimanjaro Christian Medical University College, Moshi, Tanzania; 15 Kilimanjaro Clinical Research Institute, Kilimanjaro Christian Medical Centre, Moshi, Tanzania; 16 Department of Anthropology and Development Studies, University of Johannesburg, Gauteng, South Africa

## Abstract

**Importance:**

Effective communication between patients and health care teams is essential in the health care setting for delivering optimal cancer care and increasing cancer awareness. While the significance of communication in health care is widely acknowledged, the topic is largely understudied within African settings.

**Objective:**

To assess how the medical language of cancer and oncology translates into African languages and what these translations mean within their cultural context.

**Design, Setting, and Participants:**

In this multinational survey study in Africa, health professionals, community health workers, researchers, and scientists involved in cancer care and research and traditional healers were invited to participate in an online survey on a voluntary basis through online platforms. The survey provided 16 cancer and oncologic terms used in cancer diagnosis and treatment (eg, *cancer*, *radiotherapy*) to participants, mostly health care workers, who were asked to provide these terms in their local languages (if the terms existed) followed by a direct or close translation of the meaning in English. The survey was open from February to April 2023.

**Main Outcomes and Measures:**

Patterns of meaning that recurred across languages were identified using thematic analysis of 16 English-translated terms categorized into 5 themes (neutral, negative, positive, phonetic or borrowed, and unknown).

**Results:**

A total of 107 responses (response rate was unavailable given the open and widespread distribution strategy) were collected from 32 countries spanning 44 African languages, with most participants (63 [59%]) aged 18 to 40 years; 54 (50%) were female. Translations for *cancer* were classified as phonetic or borrowed (34 [32%]), unknown (30 [28%]), neutral (24 [22%]), and negative (19 [18%]), with the latter category including universal connotations of fear, tragedy, incurability, and fatality. Similar elements connoting fear or tragedy were found in translations of terms such as *malignant*, *chronic*, and *radiotherapy*. The term *radiotherapy* yielded a high percentage of negative connotations (24 [22%]), with a prevailing theme of describing the treatment as being burned or burning with fire, heat, or electricity, which may potentially hinder treatment.

**Conclusions and Relevance:**

In this survey study of cancer communication and the translation of oncology terminology in African languages, the findings suggest that the terminology may contribute to fear, health disparities, and barriers to care and pose communication difficulties for health professionals. The results reinforce the need for culturally sensitive cancer terminology for improving cancer awareness and communication.

## Key Points

**Question:**

Does cancer care terminology exist in African languages, and if so, what do these translations mean within their cultural context?

**Findings:**

This survey study of 107 health care workers and cancer researchers revealed diverse cancer terminology in 44 African languages across 32 countries in Africa. Translations of key terms including *cancer*, *malignant*, *chronic*, and *radiotherapy* commonly conveyed elements of fear and tragedy.

**Meaning:**

The findings highlight ethno-linguistic diversity and how the language used to communicate about cancer can potentially contribute to fear, stigma, and communication challenges between patients and health care workers.

## Teaser

This survey study investigates how medical cancer and oncology terms are translated into African languages and explores what these translations mean within their cultural context.

## Introduction

An estimated 801 000 incident cancer cases and 520 000 cancer deaths occurred in Africa in 2020.^1,2^ The cancer burden in this region is increasing faster than in any other region of the world^3^; thus, cancer surveillance, research, and control programs for prevention are major priorities. Effective communication between patients and care teams is essential for empowering primary and secondary cancer prevention as well as enhancing patient engagement and treatment adherence. Despite this importance, communication has been given insufficient attention in the African context. Barriers in cancer communication can contribute to stigma and disempowerment, impacting both patients and health care workers (HCWs).^4,5,6^ Communication challenges between HCWs and patients are not confined to a specific region; they are prevalent issues globally, cutting across cultures and sociodemographic groups within a highly mobile globalized world. Health care workers face challenges in conveying information to patients due to linguistic differences.^7,8^

Language, with its transformative power, can either empower individuals to actively participate in their care or disenfranchise them, impeding their engagement with the health care system. The way cancer is discussed and how cancer communication translates in terms of meaning are important given that language intricately shapes actions, exerting influence from symptom recognition to the proactive pursuit of treatment and care. In specific cultural contexts, the linguistic portrayal of a patient’s experience with cancer and cancer care often uses metaphors of warfare and violence.^9,10^ Metaphors such as *fighting cancer*, *survivors*, and *losing the battle* are commonly used in the health care setting, patient narratives, and the broader community.^9^ Individuals with cancer are often characterized as warriors or fighters, while therapeutic interventions are metaphorically framed as weapons.^9^ The nuanced impact of these metaphors remains a subject of ongoing debate.

Globally, irrespective of the existence of a term for cancer in various cultures, the condition often carries stigma. In Western societies, such as Canada and the UK, cancer is frequently colloquially labeled as the “C-word”^11^ or “the big C,”^12^ reflecting its perceived ominous, intimidating, and sensitive nature. In India, cancer is euphemistically referred to as “a problem.” In the Netherlands, a prevalent insult involves telling someone to “get cancer.”^13^ Some academic discourse suggests refraining from using the term *cancer* for cancers classified as low risk, such as slow-growing papillary thyroid cancers, aiming to alleviate potential patient anxiety that might influence decisions toward more invasive treatments.^14^

In Africa, the absence of many scientific terms in the approximately 2000 languages spoken poses challenges in understanding medical terminology.^15,16^ Local languages may lack adequate terminology for diseases and their treatment, exacerbating challenges in accessing timely diagnosis and treatment. The present study aimed to investigate the meaning of cancer terminology in African languages and how language might contribute to fear, stigma, and communication challenges for HCWs, potentially perpetuating misconceptions and myths about the disease.

## Methods

In this survey study, we invited HCWs, community health workers, researchers, and scientists involved in cancer care and research as well as traditional healers to respond to an online questionnaire available in English, French, Portuguese, and Arabic (eAppendix in Supplement 2). The survey was developed by the International Agency for Research on Cancer (IARC) in partnership with Aga Khan University, Nairobi, and was disseminated by the African Organisation for Research and Training in Cancer, a collaborator with a vast network of HCWs and researchers in cancer care and research. The survey was also shared on the authors’ institutional platforms and networks after ethical approval was obtained by the IARC ethics committee. Written informed consent was sought from all participants at the beginning of the survey. The survey was open from February to April 2023 and received at least 1 response from most countries in Africa. The survey provided a list of cancer terms (Table 1) used in cancer diagnosis and treatment to participants, who were asked to provide each term in their local language followed by a direct translation of its meaning into English, French, Arabic, or Portuguese. Detailed methods are available in the eMethods in Supplement 2. The study followed the Consolidated Criteria for Reporting Qualitative Research (COREQ) and American Association for Public Opinion Research (AAPOR) reporting guidelines. Survey participants did not receive compensation.

**Table 1.** Summary and Thematic Analysis of 16 Terms and Translations Used in Cancer Care in African Languages

Oncology term	Responses by theme, No. (%) (N = 107)					Example (language, country)
	Neutral	Negative	Positive	Phonetic or borrowed	Unknown	
Cancer	24 (22)	19 (18)	0	34 (32)	30 (28)	Neutral physical descriptions: “abnormal growth,” “chronic illness,” “lump,” “something that proliferates” Negative connotations: “a scary thing (like a moving human skeleton)”—*kokolo* (Luganda, Uganda); “chronic illness that leads to death”—*kokolo* (Luganda, Uganda); “hunter, arrow, devouring wound, forest disease”—*gao*, *hangao*, *bi* (Djerma, Niger); “evil spirit infliction”—*migl* (Babur, Nigeria); “wound with which we will be buried”—*gomou robal* (Wolof, Senegal); “a parasitic plant”—*gomarara* (Shona, Zimbabwe); “a growing ulcer that does not heal”—*imvukuzane* (Ndebele, Zimbabwe); “destroyer”—*umdlavuzo* (Ndebele, Zimbabwe) Phonetic or borrowed: *saratane*, *saratani*, or *saratan*—Arabic for *cancer*, used in Tanzania, Kenya, Chad, Algeria, Sudan, and Morrocco; *kankere* (Bostwana); *maladi ya kansere* (DRC); *kanza* (Kenya); and *khansa* (Malawi)
Tumor	65 (61)	5 (4)	0	0	37 (35)	Neutral physical descriptions: “mass” or “swelling”—Kabyle (Algeria), seTswana (Botswana), Luganda (Uganda); “something inherited”; “swelling”; “growth”; “cyst” Negative connotations: “an incurable swelling”—*ekizimba ekitarukukira* (Rutooro, Uganda); “a growth on skin believed to be a result of having been bewitched”—*chitsinga* (Shona, Zimbabwe); “devouring wound”—*bi ngnoiri* (Djerma, Niger); “destroyer”—*umdlavuzo* (Ndebele, Zimbabwe)
Malignant	34 (32)	30 (28)	0	0	43 (40)	Neutral physical descriptions: “replicates,” “spread,” “growth that carries cancer cells in it,” “advanced or escalated” Negative connotations: “malevolent or evil”—*balawou* (Djerma, Niger); “deadly or destructive swollen”—*wiwu tin se jamba* (Yoruba, Nigeria); “with serious consequences”—*ya mbano ya mabe* (Lingala, DRC); “bad or severe”—*djuman* (Malinke, Guinea); “it will kill”—*na gi oni* (Ogbia, Nigeria); “it can kill”—*kougou* (Kotokoli, Togo); “it kills”—*ewounamé* (Éwé, Togo); “angry or intending to do harm” or “of dangerous or angry nature”—*kwaadaardig* (Afrikaans, South Africa); “without healing”—*kougou* (Kotokoli, Togo)
Radiotherapy	43 (40)	24 (22)	0	0	40 (37)	Neutral physical descriptions: “cancer treatment using high energy x-rays”—*kurapa gomarara nema* x-ray (Shona, Zimbabwe); “using light in searching for a hidden object”—*kuvhenekwa* (Shona, Zimbabwe); “to be treated with special light”—*ho alafuoa ka lebone le ikhethileng* (Sotho, Lesotho); “radioactive waves emitted to an affected part to treat or control cancer spread”—*marang a a dirisiwang go lwantsha* or *alafa kankere* (seTswana, Bostwana); “heal using energy”—*go alaha ka motlakase* (seTswana, Botswana); “to light up”—*go bonesa* (seTswana, Botswana) Negative connotation using *burn*: “burning”—*kuwotchelela* (Chichewa, Malawi); “burn”—*ukushiswa* (Zulu, South Africa) and *ukutshiswa* or *ukutshisa* (Ndebele, Zimbabwe); “burn”—*kupisiwa* (Shona, Zimbabwe); “burn”—*kushoka* (Ngoni, Zambia); “searing the tumor”— الكي (Arabic, Algeria); “to be burnt” or “burning treatment”—*kuchomwa* or *matibabu ya kuchoma* (Swahili, Kenya); “treatment by burning”—*kalahi ka go tshuba* (seTswana, Bostwana); “treatment that burns cancer”—*wanko dyankaro dyenin basi* (Malinke, Guinea); “burning treatment” or “to burn”—*brand* or *om te brand* (Afrikaans, Namibia); “light to burn cancer cells” (English, Cameroon); “fire treatment”—*bugum tibegɔ* (Gurene, Ghana) Negative connotations using *electricity* or *heat: ugesi* (Xhosa, South Africa); *masanyalaze* or *amashanyaraze* (Luganda, Uganda); *kukalirira* (Luganda, Uganda); *électricité* (Kabyle, Algeria); “heating”— تسخين (Arabic, Libya); “warming”—التسخين (Arabic—Libyan dialect, Libya)
Chemotherapy	70 (65	2 (2)	0	0	35 (33)	Neutral physical descriptions: “medication for cancer,” “cancer treatment” Negative connotations: “burn”—*go tshuba* (seTswana, Botswana); “poison”—*gif* (Afrikaans, Namibia) Positive connotation: “to heal”—*go alaha* (seTswana, Botswana)
Benign	29 (27)	3 (3)	34 (32)	0	41 (38)	Neutral physical descriptions: “does not reproduce,” “not cancerous” Positive connotations: “good or foolish”—*bego* or *jil* (Ahmaric, Ethiopia); “without serious consequences”—*ya mbano ya makasi te* (Lingala, DRC); “friendly”—*onskadelik* (Afrikaans, Namibia); “it will not kill or cause death”—*orusa gi oni* (Ogbia, Nigeria) (similar translations reported in Togo in the Éwé, Kotokoli, and Mina languages); “kind”—*hamida* (Arabic, Sudan); “harmless,” “no harm,” “not harmful”—various translations recorded from participants in Malawi (Chichewa), Mozambique (Portuguese), Mali (Bambara), Namibia (Afrikaans), Niger (Djerma), Nigeria (Hausa), South Africa (seTswana, Xhosa), Uganda (Rutooro), Zimbabwe (Shona) Negative connotation: “a bulge caused by dirty blood”—*uvimbe*, *mtoki*, or *jipu* (Swahili, Tanzania)
Biopsy	67 (63)	2 (2)	0	0	38 (35)	Neutral physical descriptions: “removing tissue for examination,” piece of flesh,” “ a lab test that involves cutting a piece of a swelling” Negative connotations: “pus”—*megil* (Ahmaric, Ethiopia); “cut tissue and cook”—*mwaa iŋa nΕnɔ dugΕ* (Gurene, Ghana)
Chronic	62 (58)	10 (9)	0	0	35 (33)	Neutral physical descriptions: common translations included “persistent disease,” “prolonged illness” Negative connotations: “incurable”—*go tlhoka kalafi* (seTswana, Bostwana); “bedridden”—*go gatellwa* (seTswana, Bostwana); “not curable”—*bolwetsi go bo sa alafiweng* (seTswana, Bostwana); “noncurative”—*go sena pholo* (seTswana, Bostwana); “incurable disease”—*yawer3 kuankro* (Twi and Gurune, Ghana); similar translations of “incurable” were reported from Uganda (Alur and Luganda), Nigeria (Igbo), and Kenya (Swahili)
Metastasis	70 (65)	1 (1)	0	0	36 (34)	Neutral physical description: “the mother mass has sent seedlings into another site”—*ekiziba kyasindika obwana bwayo ahare* (Luganda, Uganda) Negative connotation: “tear up”—*umdlavuza* (Zulu, South Africa)
Staging	64 (60)	0	0	1 (1)	42 (39)	Borrowed or phonetic: “staging”—*di stage tsa kankere* (seTswana, Botswana) Explanation: “stage” is also borrowed from the English term for phonetic purposes
Surgery	24 (22)	19 (18)	0	34 (32)	30 (28)	Neutral physical description: “to put right or to tidy up”—*okulongosa* (Luganda, Uganda) Negative connotations: “to be butchered” or “butchered” (kiKamba, Kenya); “at a knife operation” (seSotho, Lesotho) Phonetic or borrowed: “operation” (multiple countries)
Trial	65 (61)	0	0	0	42 (39)	Neutral physical descriptions: translations were mainly related to treatment testing, research, and investigation, such as “clinical research,” “medical investigation,” and “clinical testing”
Palliation	60 (56)	1 (1)	0	0	46 (43)	Neutral physical descriptions: most translations alluded to concepts such as “comfort care,” “home-based care,” and “supportive care”; “making bearable”—*ukulalisa* (Ndebele, Zimbabwe) Negative connotation: “hopeless treatment”—*kongeri tibegɔ* (Gurene, Ghana)
Recur	84 (79)	0	0	0	23 (22)	Neutral physical descriptions: most translations within this theme involved references to the disease returning or coming back, such as “cancer has returned,” “to resurface after it has been destroyed,” or “relapse”
Survival	43 (40)	0	41 (38)	0	23 (22)	Positive connotations: “healed” or “healing,” “achievement,” “to win,” “overcome the disease,” “living,” and “to be saved” Positive connotations with metaphors of warfare: “to defeat a life-threatening situation,” “to conquer”
Remission	25 (23)	0	40 (38)	0	42 (39)	Positive connotations: similar to the term *survival*, the translation “heal” or “healing” was common; other translations included “to get better,” “disappearance or reduction of symptoms,” “recover,” “lessening of the sickness,” “leave or forgive,” “to set free from,” “to forgive,” “being forgiven,” or “I am clean”

Abbreviation: DRC, Democratic Republic of the Congo.

### Thematic Analysis

We conducted thematic analysis of the English-translated terms to identify patterns of meaning that recurred across languages. The analysis followed a 6-step process of familiarization, coding, generating themes, reviewing themes, defining and naming themes, and writing and adaptation of the COREQ guideline.^17,18^ This analysis identified 5 themes, into which the responses for all 16 terms (*cancer*, *tumor*, *malignant*, *radiotherapy*, *chemotherapy*, *benign*, *biopsy*, *chronic*, *metastasis*, *staging*, *surgery*, *trial*, *palliation*, *recur*, *survival*, and *remission*) were categorized: (1) *neutral* was used for translations that offered outcome-based or descriptive explanations of the term, maintaining a neutral and factual tone; (2) *negative* was used to describe translations with negative connotations, outcomes, or emotions that superseded the neutral description; (3) *positive* was used to describe translations with positive connotations, outcomes, or emotions that superseded the neutral description; (4) *phonetic or borrowed* was used to describe terms that had phonemic semblance to the original term or for terms that were borrowed from another language; and (5) *unknown* was used for unknown or nonexistent translations.

### Statistical Analysis

Descriptive statistical methods were used to analyze the survey data using Stata, version 17 (StataCorp LLC). Quantitative analysis involved calculating the frequency and percentage of responses within each theme as well as summary statistics for age, self-reported gender, and profession distribution. No advanced inferential statistics were used.

## Results

**Figure 1.**
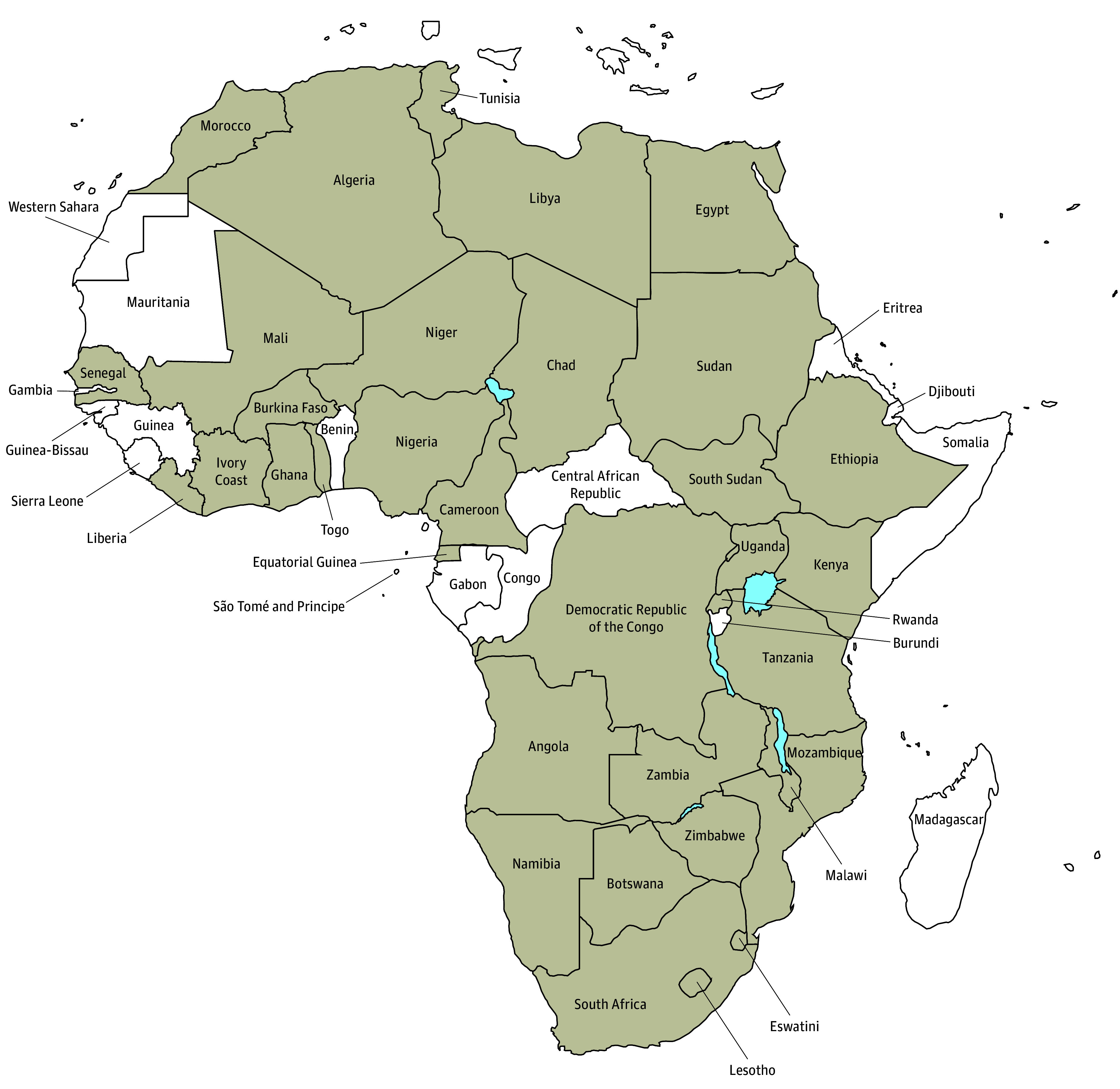
Countries Where Participants Provided Cancer Terms and Translation Shading indicates countries that provided terms and translation. The diagram was created with MapChart.

A total of 107 responses were received and were equally distributed between participants identifying as men (53 [49%]) and women (54 [50%]), with 32 (58%) of a total of 55 African countries represented (eTable in Supplement 1). The response rate was not calculated given the open and widespread distribution strategy. Most participants (63 [59%]) were aged 18 to 40 years followed by 38 (36%) aged 41 to 60 years and 6 (6%) aged 60 years or older. The study’s participants were predominantly HCWs (62 [58%]), with 32 (30%) specializing in oncology and 30 (28%) in other health care fields. Cancer researchers (n = 31) composed 29% of the sample, while researchers from other disciplines (n = 7) constituted 7%. Seven participants (7%) represented diverse professions, including students and religious leaders. The largest number of participants was from Nigeria (11 [10%]) followed by South Africa (9 [8%]). The geographic coverage of this survey is shown in Figure 1. A total of 44 languages were reported, with the most reported languages being Arabic (11 [25%]), Swahili (7 [16%]), Afrikaans (6 [14%]), and Amharic (6 [14%]) (eFigure in Supplement 2). Many of these languages originated from multiple countries, such as Arabic (Algeria, Egypt, Libya, Morrocco, Sudan, Chad, and Tunisia), Swahili (Kenya, Uganda, and Tanzania), Tswana (South Africa and Botswana), and Afrikaans (Namibia and South Africa).

An overview of participants’ responses across all 16 terms revealed a consistent pattern, with 23 (22%) and 46 (43%) indicating that the terms *survival* and *palliation*, respectively, either did not exist in their language or that they were unsure of the translations; the remaining terms fell within this range (Table 1). Some terms stood out with a higher percentage of negative connotations, such as *cancer* (19 respondents [18%]), with translations such as “evil spirit infliction,” “chronic illness that leads to death,” and “destroyer”; *surgery* (19 [18%]), with translations including “to be butchered”; and *malignant* (30 [28%]), with translations such as “malevolent or evil” and “angry or intending to do harm.” Another term with a relatively high percentage of translations coded as negative was *radiotherapy* (24 [22%]). Additionally, for the term *chronic*, a notable proportion of participants (10 [9%]) expressed negative perceptions through translations such as “incurable” and “bedridden.”

### Cancer

Seventy-seven participants (72%) recorded a term for cancer that existed in their local vernaculars. The themes established for these lexical translations encompassed distinct classifications: phonetic or borrowed (34 [32%]), neutral (24 [22%]), negative (19 [18%]), and an overarching category of unknown (30 [28%]) (Table 1).

Participants from Tanzania, Kenya, Chad, Algeria, Sudan, and Morrocco reported that the term for cancer in their respective languages was *saratane*, *saratani*, or *saratan*. These terms are borrowed from the Arabic lexeme *saratan*, which means “cancer.” This etymological borrowing underpins the relationship between these linguistic expressions, as they share a common origin in their historical roots. Regarding the theme “phonetic or borrowed,” 10 terms that phonemically resemble the term *cancer* were reported, which included *kankere* (Botswana), *maladi ya kansere* (Democratic Republic of Congo), *kanza* (Kenya), *khansa* (Malawi), *oria kansa* or *kansa* (Nigeria), *kansa* (Tanzania), *kanseri* (Rwanda), *kanker* (South Africa), and *kansevi* (Togo). Within the thematic category “negative” were a series of translated terms that evoked fear or suggested the incurable nature of cancer and translations laden with malevolent spiritual connotations.

The term *cancer* in Luganda (language spoken in Uganda) was reported by participants to be *kokolo* or *kookolo*, and 1 participant elaborated,

The term *kookolo* has come to directly translate to cancer, although it is not known by everyone. Many members of the general public do not know names of diseases until they have directly been affected by them. Other terms used in place of *cancer* are descriptive terms such as “obulwadde bw’ebizimba,” which means “illness (characterized) of swellings,” or “obuladde bw’ebbwa eritawona,” which means “illness of a wound that does not heal.”

In Shona, a language spoken in Zimbabwe, the term is *gomarara*, which means a parasitic plant. A participant explained, “This is a plant that grows on top of another plant, in a parasitic way, usually killing or disabling the plant.”

### Radiotherapy

For the term *radiotherapy*, 67 participants (63%) reported that the term existed in their local language. The themes established for these etymological translations were neutral (43 [40%]), negative (24 [22%]), and unknown (40 [37%]). The term *radiotherapy* yieled a high percentage of translations with negative connotations (Table 1), with references to burning, roasting, or being burned with fire, heat, and electricity. Exemplifying this category, a participant from Uganda who speaks Luganda remarked,

The scientific words “radiation therapy” are translated in our local language as “roasting” or using “electricity.” In the actual sense, some of the radiotherapy side effects are the dry and moist skin desquamation, which physically appear like burns or partially roasted meat. So to a layperson or a patient who is already anxious, this is “Gospel truth.” During health education, some clients will ask, “Are we going to be put in the machine (direct English translation) and be roasted like meat?” The one roasting is the radiation therapist and the local oven is the Cobalt-60 machine. The stalks of meat are the parts on the patient’s body to be radiated.

**Figure 2.**
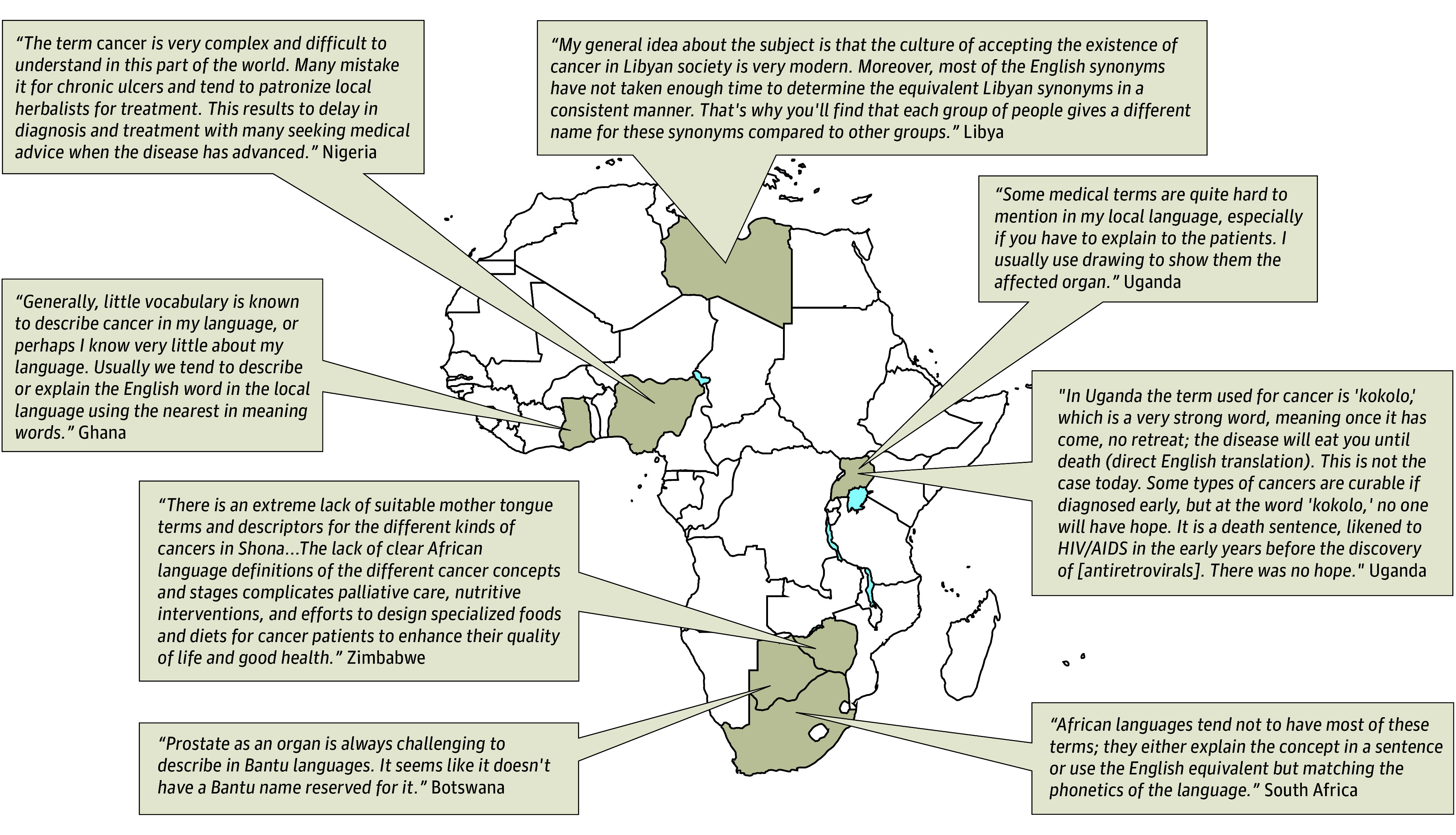
Selected Participants’ Insights for Botswana, Ghana, Libya, Nigeria, Uganda, South Africa, and Zimbabwe

Participants also shared perspectives on the overarching theme of translating cancer terms into local languages (Figure 2) and cited reasons for communication challenges (Box).

Box. **Select Health Care Workers’ Responses to Whether They Experienced Challenges or Barriers Communicating With Patients Using the Local Language**

**Responses**

“Yes, especially when dealing with [elderly patients] who have never been to school.” Botswana (seTswana)
“Sometimes. I navigate by explaining where the organ is located, its function, and then probing the understanding of the patient.” Botswana (seTswana)
“Principal barriers are illiteracy.” Guinea (Toma)
“Barriers or challenges are encountered when communicating cancer terms, but explaining in broader terms in the local language usually helps.” Malawi (Chichewa)
“Yes. I have downloaded applications to help me with translations; however, they are not always accurate.” South Africa (isiZulu)
“Easily understood, but the etiology and more info usually are needed as patients do not know more details about what exactly they suffer from.” Tanzania (Kiswahili)
“Since the whole cancer concept is new to many patients, and they are more familiar with infectious diseases, it can take time for patients/caregivers to grasp concepts. Repeated communication and giving illustrative examples is crucial.” Uganda (Luganda)
“Some words just don’t exist.” Zambia (Ngoni)

## Discussion

### Summary

This study investigated the symbolic relevance and importance of cancer terminology in African populations and how these understandings impact communication efforts in care and practice in health care settings in Africa. Results for the 16 terms used in cancer care revealed a spectrum of diversity in terminology and translations. Participants’ local terms often contained linguistic references reflective of their cultural and social contexts. Our study highlighted the nonexistence or lack of knowledge of cancer terminology in African languages for approximately one-third of respondents for a given term. The revelation that certain terms may not exist or are not widely enfolded into lexicons of meaning in some African languages points to potential barriers in disseminating crucial information about cancer and its care. The results highlight the need for patient-centered communication strategies, HCW training, and engaging patients with cancer and cancer survivors to integrate their lived experiences.

### Cancer Translations Needing Attention

In our study, the term *cancer* had translations with connotations of fear, tragedy, incurability, and fatality reported from several languages and countries. Certain terms and their translations incorporated malevolent spiritual undertones, indicating a connection between perceptions of illness and belief in spiritual causation. The weightiness associated with the term *cancer* often extends to its connotation of being overwhelming, unbeatable, and frequently final, contributing to a sense of cancer fatalism. This connotation was evident in our results from the terms used for *cancer* in several countries, underscoring the gravity and inevitability of fatality.

The term *radiotherapy* invoked ideas across several languages that referenced fire, heat, and electricity. A prevalent theme emerged wherein *radiotherapy* was referred to by HCWs and researchers as burning, roasting, or being burned. This recurrent motif emphasizes a widespread conceptualization of radiotherapy rooted in the elemental forces of fire and heat across diverse linguistic contexts. This raises concerns about the potential psychological effect on patients, as it may induce fear and apprehension and may be a deterrent to treatment initiation. While this characterization may initially evoke concerns, questions remain regarding whether radiotherapy can be reframed positively as a symbol of the transformative and healing nature of the treatment. Negative connotations for radiotherapy are not unique to African languages; in many European languages, the term *radiotherapy* is often similar to the term used in nuclear accidents.

The translations provided for the term *chronic* revealed a concerning divergence from its true definition. This discrepancy highlights the need for accuracy and clarity when conveying the concept of chronicity. Of note is the 9% of translations with negative connotations, with expressions such as “incurable,” “bedridden,” and “noncurative.” These translations present a notable departure from the medical connotation of chronic conditions, which generally denote long-term persistence^19^ rather than an inherent lack of cure or immobility. These translations emphasize a critical need for medical-linguistic precision to accurately convey the medical concept of chronic conditions.

The findings of our study showed that some participants reported the nonexistence of various cancer-related terms in their languages or expressed uncertainty, ranging from 23 (22%) for terms like *survival* and *recur* to 46 (43%) for *palliation*. This highlights potential challenges in conveying essential cancer concepts. For instance, a participant noted the absence of the word *prostate* in Bantu languages, illustrating challenges in explaining a common cause of cancer mortality in African men.

### Richness of Translations

The translation for the term *metastasis* provided by a Ugandan participant, “ekiziba kyasindika obwana bwayo ahare,” meaning “the mother mass has sent seedlings into another site,” is particularly noteworthy as it exemplifies a unique linguistic expression deeply rooted in idioms and proverbs within the Luganda language. This intricate metaphorical construction, likening metastasis to the dispersal of seedlings from a mother mass, underscores the richness of cultural idioms embedded in African languages to describe complex medical concepts. It reveals an interplay between traditional linguistic expressions and the articulation of scientific terms, emphasizing the need to recognize and appreciate the diverse and metaphorical nature of cancer terminology within African linguistic and cultural contexts.

In terms of idioms of distress^20^ used, exemplified in instances where cancer was referred to as the “wound with which we will be buried” (translated from Wolof), these embedded meanings signify psychological and emotional markers of distress related to the participants’ culturally located experiences and perceptions of cancer. Other linguistic expressions, like “forest disease” (translated from Djerma) and “parasitic plant” (translated from Shona), highlight the wider symbolic import of where and how cancer is understood differently across geographies and cultures.

The interpretations of *survival* and *remission* in translations carried positive undertones, suggesting notions of healing, cure, and recovery. In certain translations for *survival*, metaphors related to warfare surfaced, such as “to defeat a life-threatening situation” and “to conquer.” For the term *remission*, a handful of translations incorporated the term *forgive*, potentially indicating a perspective that views cancer as a form of punishment.

### Comparisons With Previous Work

Work done in South Africa with patients with cancer highlighted that the term *cancer* exists in only 3 of 11 official languages: isiZulu, siSwati, and isiXhosa (Umdlavuza, U’mdlopha, and Umlaza, respectively).^21^ Evidence from a 2016 Kenyan study suggests that language problems may hinder patients and family members from comprehending cancer diagnoses and seeking out appropriate interventions.^5^ A South African study analyzing perspectives on cancer, chemotherapy, and radiotherapy among 9 Xhosa-speaking patients with cancer revealed an overall lack of knowledge about cancer and cancer treatment.^22^ An added complexity in the African setting is that patient-centered communication and treatment decision-making may not be directly communicated to the persons with cancer themselves, especially for older persons.^5,23^ The challenge of effective communication in cancer care is not unique to Africa. A report from the US emphasized that inadequate patient-practitioner communication was a significant factor contributing to the suboptimal state of cancer care delivery.^24,25^

The challenges linked with health communication extend beyond cancer to other medical conditions such as tuberculosis, HIV infection and AIDS, and mental illness.^26,27^ Tuberculosis is frequently labeled as the “disease of poverty,” which stigmatizes those affected.^26^ In tuberculosis care and research, certain terms, shared with the context of cancer, contribute to stigmatization by embodying “metaphors of transgression and punishment.”^26^ Noteworthy examples include *treatment defaulter*, implying a judgment akin to nonpayment of a loan; *tuberculosis suspect*, suggesting criminal behavior; and *noncompliant*, assigning blame and patient labeling without acknowledging systemic and structural barriers to treatment adherence.^26^ In the context of cancer, terms such as *delayed presentation* or *loss to follow-up* may erroneously suggest patient fault, overlooking the repeated engagements individuals experiencing delays in diagnosis have with the health system, which are common in low- and middle-income countries.^28,29^ Work done during the early years of the HIV epidemic in Africa to overcome stigmatizing language also serves as a blueprint for cancer communication.^30,31,32^ Initiatives such as the Stop TB Partnership’s Tuberculosis Language Guide have been instrumental in outlining nonstigmatizing alternatives for tuberculosis care and research, which may lend lessons to the oncology setting.^26^ The American Cancer Society’s initiatives for patient and caregiver education and the International Atomic Energy Agency’s Rays of Hope radiotherapy program show the potential for translations^33^ and positive terminology alternatives^34^ in Africa.

### Limitations

This study has limitations. While the insights from this study offer valuable contributions to our understanding of the linguistic nuances associated with cancer treatments, caution must be exercised in generalizing the findings. The study’s small sample size based on convenience sampling poses limitations, and the use of an online survey as the primary data collection tool may not have captured the depth required for a comprehensive analysis but provided initial insightful information. Another limitation is the lack of patient perspectives in our study. Our focus on HCWs and researchers trained in the language of Western medicine may not have fully captured the linguistic nuances experienced by patients. Recommendations for future work are detailed in Table 2.

**Table 2.** Recommendations for Future Studies

Recommendation	Description
Expand to patient perspectives	Include patient perspectives in future studies to gain a broader understanding of how cancer terminology affects their experiences, emotions, and treatment decisions.
Conduct in-depth country analyses	Use in-depth interviews locally within countries to gather richer insights and allow participants to fully express their views.
Develop alternative terminology	Establish think tanks to explore how to communicate potentially fear-inducing terms like *radiotherapy* or how to develop alternative terminology for terms that invoke fear or stigma or disempower patients. A focus on local ownership in development of solutions is needed.
Investigate mediated messaging impact	Study how indirect communication (where information is conveyed to family members rather than directly to patients) affects cancer care.
Standardize terminology	Work toward standardizing cancer terminology across different languages to reduce confusion and improve communication in health care settings.
Evaluate educational programs	Assess the effectiveness of educational programs designed to inform patients and communities about cancer to reduce stigma and fear associated with the disease.
Collaborate with local communities	Engage local communities and cultural leaders in the development of cancer communication strategies to ensure they are culturally sensitive and effective.

## Conclusions

The findings of this survey study showed that oncology terminology in African languages may contribute to fear, health disparities, and barriers to care and create communication difficulties for health professionals. There is a pressing need for the development of alternatives to cancer terms that invoke fear or stigma or disempower patients in local languages. Multisectoral approaches between clinicians, anthropologists, and communities may help to develop an emerging lexicon that offers neutrality or at least hope of treatment and/or cure. This study highlights the need for culturally sensitive approaches to cancer communication in health care settings across African populations.

## Notes

Supplement 1. **eTable.** Characteristics of Survey Participants and Responses

Supplement 2. **eMethods.** Comprehensive Methods for the Study **eFigure.** Country Origins of Participants and Their Respective Languages **eAppendix.** Survey

Supplement 3.
Data Sharing Statement
