# Supplementary material for: Cancer Care Terminology in African Languages
Source: JAMA Netw Open. 2024 Aug 30;7(8):e2431128. doi: 10.1001/jamanetworkopen.2024.31128 (PMC11364996; doi:10.1001/jamanetworkopen.2024.31128)
Supplement: Supplement 2. — eMethods. Comprehensive Methods for the Study eFigure. Country Origins of Participants and Their Respective Languages eAppendix. Survey [file jamanetwopen-e2431128-s002.pdf]

Supplementary Online Content

Simba H, Mutebi M, Galukande M, et al. Cancer care terminology in African languages. *JAMA Netw Open*. 2024;7(8):e2431128. doi:10.1001/jamanetworkopen.2024.31128

eMethods. Comprehensive Methods for the Study

eFigure. Country Origins of Participants and Their Respective Languages

eAppendix. Survey

This supplementary material has been provided by the authors to give readers additional information about their work.

© 2024 Simba H et al. *JAMA Network Open*.

eMethods: Comprehensive Methods for the study

The survey was co - developed by researchers at IARC in collaboration with Aga Khan and University of Johannesburg researchers. The majority of the researchers involved in this process were African. We invited health professionals, community health workers, researchers and scientists involved in cancer care/research, and traditional healers to respond to an online questionnaire (google form) available in English, French, Portuguese, and Arabic. The survey was piloted to the entire of the team as well as nurses from Uganda. The survey collected responses using purposive and convenience sampling online, and was pre-tested and piloted for clarity. Back translation of the Arabic, Portuguese and French Questionnaires was done by team members. The survey was open from February to April 2023, having received at least one response from most countries.

The survey provided a list of cancer terms used in cancer diagnosis and treatment to participants, who were asked to provide each term in their local language followed by a direct translation of its meaning into English, French, Arabic and Portuguese. The list of 16 terms included cancer, tumour, biopsy, malignant, benign, staging, metastasis, surgery, chemotherapy, radiotherapy, trial, remission, palliative care, survival, recur, and chronic. Information on the participant’s country, name of language, age group, profession and gender identity were provided, including HCWs confidence in communicating with patients in their local language.

Our study adhered to American Association for Public Opinion Research (AAPOR) reporting guidelines to ensure transparency and reliability. The survey collected

© 2024 Simba H et al. *JAMA Network Open*.

responses using purposive and convenience sampling online, and was pre-tested and piloted for clarity. Descriptive statistical methods and thematic analysis were conducted. We disclosed potential biases and limitations. No weighting was applied as the study aimed to capture a diverse range of linguistic and cultural interpretations rather than producing population estimates. We commit to transparency by providing access to the survey instrument and data collection details upon request, ensuring the study's findings are replicable and reliable.

Descriptive statistical methods were used to analyze the survey data using STATA version 17. Quantitative analysis involved calculating the frequency and percentage of responses within each theme as well as summary statistics for the age, gender and profession distribution. No advanced inferential statistics were applied.

A COREQ reference has been added in text. In summary, the coding and categorization of survey responses were conducted through a systematic and collaborative process. A team of three investigators reviewed all responses independently to ensure accuracy and consistency in assigning categories (neutral, negative, etc.). Each response was initially reviewed and coded by two investigators independently. In cases where there were discordant interpretations, a third investigator reviewed the responses to resolve discrepancies. The final categorization was achieved through consensus among the investigators.

Coding and theme development were conducted by the same team of investigators, allowing for a cohesive understanding of the data and ensuring consistency throughout the analysis. This collaborative approach helped to maintain objectivity and reliability in the categorization process. The coding and theme development was then presented to the entire authorship team during a meeting.

© 2024 Simba H et al. *JAMA Network Open*.

eFigure: Country origins of participants and their respective languages. The different colour codes uniquely represent each country included in the survey, along with the languages reported by participants from those countries.

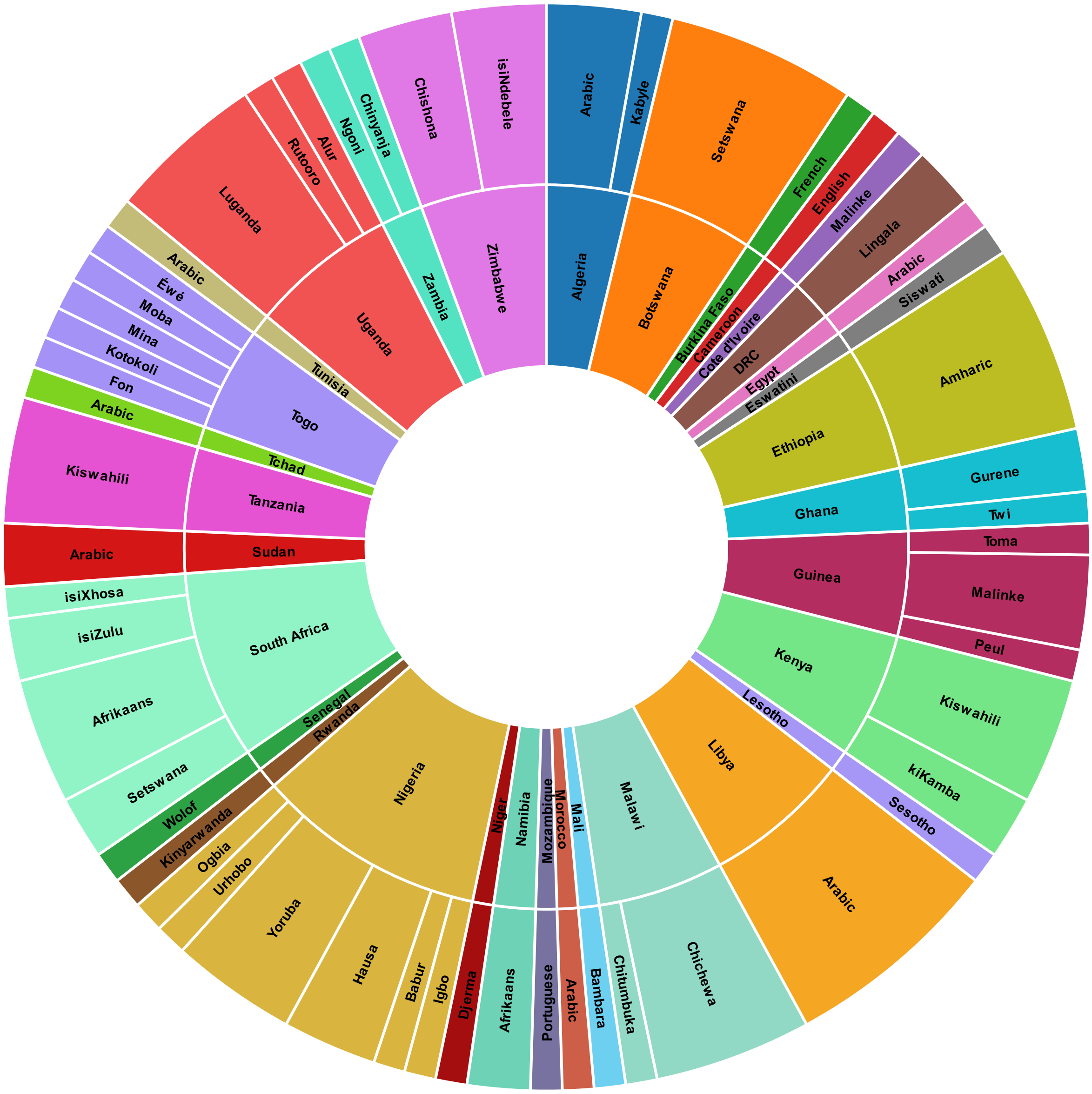

© 2024 Simba H et al. *JAMA Network Open*.

eAppendix: Survey (Google form)

The Language of CancerCommunication in Africa

Dear Participant,
Thank you for taking the time to read and participate in this survey. We provide a brief introduction here and the survey follows.
At the International Agency of Research in Cancer (IARC/WHO) and in collaboration with the African Organisation for Research and Training in Cancer (AORTIC), we are conducting a survey on "The Language of Cancer Communication in Africa". The aim herein is to gather information on how the medical language of cancer and its treatment is communicated to patients and in the community in African languages, and what these communication terms mean. The study will highlight the existence or nonexistence of cancer terms as well as the nature of cancer terminology in African languages and how it may contribute to fear, disparities for patients, and pose communication difficulties for Health Professionals. The survey will collect information on cancer terms translated to African languages. You will be asked to translate selected cancer terms, (mostly those used in diagnosis and treatment) into your African language, and then directly translate them back into English.
We are inviting all health professionals, community health workers, researchers and scientists involved in cancer care, to participate in this study. We hope to capture all African countries and all of the major languages for each country, thus we invite you to share the survey link with other relevant professionals and especially those from other countries.
We envision that information collected from this survey will highlight the state of cancer communication in Africa, and serve as platform and basis for future work on the topic. The information collected in this survey will be analysed and published in a manuscript.
You can take this survey once for each language you speak.
Thank you for your time and all information provided. If you would like to provide or obtain further information, please email Dr Hannah Simba (simbah@iarc.who.int)
Dr Hannah Simba, https://www.iarc.who.int/staff_member/hannah-simba/
Branch of Environment and Lifestyle Epidemiology, International Agency for Research on Cancer
On behalf of Dr Miriam Mutebi, Dr Moses Galukande, Dr Adamu Addissie, Dr Efua Prah, and Dr Valerie McCormack (IARC)

The Language of Cancer Communication in Africa

* Indicates required question

© 2024 Simba H et al. *JAMA Network Open*.

Consent form
The purpose of this research project is to collect information on on how cancer terms are communicated to patients and in the community in African languages. This is a research project being conducted by the International Agency of Research in Cancer (IARC/WHO) in collaboration with AORTIC. You are invited to participate in this research project because you are a health professional working in cancer care, or a cancer researcher.
Your participation in this research study is voluntary. You may choose not to participate. If you decide to participate in this research survey, you may withdraw at any time. If you decide not to participate in this study or if you withdraw from participating at any time, you will not be penalized.
The procedure involves filling an online survey that will take approximately 30 minutes. Your responses will be confidential, and we do not collect identifying information such as your name, email address or IP address. The survey questions will be about translations of cancer terms used in diagnosis and treatment in African languages
We will do our best to keep your information confidential. All data is stored in a password protected electronic format. To help protect your confidentiality, the surveys will not contain information that will personally identify you. The results of this study will be used for scholarly purposes only.
If you have any questions about the research study, please contact Dr Hannah Simba (simbah@iarc.who.int).
1.

ELECTRONIC CONSENT: Please select your choice below.Clicking on the "agree" button below indicates that:• you have read the above information• you voluntarily agree to participate• you are at least 18 years of ageIf you do not wish to participate in the research study, please declineparticipation by clicking on the "disagree" button.
*
Check all that apply.
Agree

Disagree

Demographics

© 2024 Simba H et al. *JAMA Network Open*.

2.

You will be asked to complete one form for each language. Which country is yourexperience from?
Mark only one oval.
Algeria
Angola
Benin
Botswana
Burkina Faso
Burundi
Cabo Verde
Cameroon
Central African Republic (CAR)
Chad
Comoros
Congo Democratic Republic of the
Congo Republic of the
Cote d'Ivoire
Djibouti
Egypt
Equatorial Guinea
Eritrea
Eswatini
Ethiopia
Gabon
Gambia
Ghana
Guinea
Guinea-Bissau
Kenya
Lesotho
Liberia
Libya
Madagascar
Malawi
Mali
Mauritania
Mauritius
Morocco
Mozambique
Namibia
Niger
Nigeria
Rwanda
Sao Tome and Principe
Senegal
Seychelles
Sierra Leone
Somalia
South Africa

© 2024 Simba H et al. *JAMA Network Open*.

South Sudan
Sudan
Tanzania
Togo
Tunisia
Uganda
Zambia
Zimbabwe
3.

How old are you?
*
Mark only one oval.
18-40
41-60
60+

4.

What is your gender identity?
Select all that apply
*
Check all that apply.
Woman

Man

Non-binary/non-conforming

Transgender

Prefer not to say

Other:

5.

Who are you?
*
Mark only one oval.
Oncologist
Oncology Nurse
Community Health Worker
Cancer Researcher
Traditional Healer

Other:
6.

What is the name of your language?
*

© 2024 Simba H et al. *JAMA Network Open*.

7.

If applicable, which region in your country does your language belong to?

Translation and explanation of cancer terms

In this section, we will ask you to translate selected cancer terms to your own language and then directly to English (if a translation exists). Please use the space provided for any additional explanations or comments regarding the translated term and its meaning.If you do not know the translation or if a term does not exist in your language, you can leave the answer blank and move to the next term.

8.

What is "Cancer" called in your language?

9.

What is the nearest English translation of this term?

Use the space provided to give any additional information that you would like us toknow regarding the meaning of this term.

10.

What is "Tumour" called in your language?

11.

What is the nearest English translation of this term?

Use the space provided to give any additional information that you would like usto know regarding the meaning of this term.

12.

What is "Benign" called in your language?

13.

What is the nearest English translation of this term?

Use the space provided to give any additional information that you would like usto know regarding the meaning of this term.

14.

What is "Malignant" called in your language?

15.

What is the nearest English translation of this term?

Use the space provided to give any additional information that you would like usto know regarding the meaning of this term.

16.

What is "Biopsy" called in your language?

17.

What is the nearest English translation of this term?

© 2024 Simba H et al. *JAMA Network Open*.

Use the space provided to give any additional information that you would like usto know regarding the meaning of this term.
18.

What is "Chronic" called in your language?
19.

What is the nearest English translation of this term?
Use the space provided to give any additional information that you would like usto know regarding the meaning of this term.
20.

What is "Metastasis" called in your language?
21.

What is the nearest English translation of this term?
Use the space provided to give any additional information that you would like usto know regarding the meaning of this term.

22.

What is "Chemotherapy" called in your language?
23.

What is the nearest English translation of this term?
Use the space provided to give any additional information that you would like usto know regarding the meaning of this term.
24.

What is "Radiation or Radiotherapy" called in your language?
25.

What is the nearest English translation of this term?
Use the space provided to give any additional information that you would like usto know regarding the meaning of this term.
26.

What is "Cancer staging" called in your language?

27.

What is the nearest English translation of this term?
Use the space provided to give any additional information that you would like usto know regarding the meaning of this term.
28.

What is "Surgery" called in your language?
29.

What is the nearest English translation of this term?

© 2024 Simba H et al. *JAMA Network Open*.

Use the space provided to give any additional information that you would like usto know regarding the meaning of this term.
30.

What is "Clinical trial" called in your language?
31.

What is the nearest English translation of this term?
Use the space provided to give any additional information that you would like usto know regarding the meaning of this term.

32.

What is "Palliation or Palliative care" called in your language?
33.

What is the nearest English translation of this term?
Use the space provided to give any additional information that you would like usto know regarding the meaning of this term.
34.

What is "Recurrence" called in your language?
35.

What is the nearest English translation of this term?
Use the space provided to give any additional information that you would like usto know regarding the meaning of this term.
36.

What is "Survival" called in your language?

37.

What is the nearest English translation of this term?
Use the space provided to give any additional information that you would like usto know regarding the meaning of this term.
38.

What is "Remission" called in your language?
39.

What is the nearest English translation of this term?
Use the space provided to give any additional information that you would like usto know regarding the meaning of this term.

Translation of cancer types
In this section we will ask you to provide names of cancer types that exist in your language
40.

© 2024 Simba H et al. *JAMA Network Open*.

If there are any cancer types (sites) that have specific, interesting or challengingtranslations in your language, please list them here in the following format: cancer name in English - cancer name in your language

41.

Do you have any additional thoughts, comments regarding this topic?

This section is for Health Professionals who interact with cancer patients, forparticipants who do not fit this category, click next

42.

When interacting with patients, do you communicate these cancer terms in yourLocal Language or in English?

43.

How confident are you in communicating these terms in your local language

Mark only one oval.

Strongly confident
Confident
Not confident
Not applicable

44.

Do you encounter any barriers or challenges when communicating these cancer terms, and how do you navigate them?

Thank you and contact

Thank you for taking the time to complete the survey. If you would like to participate further in this study, please send us an email. Please email Dr Hannah Simba (simbah@iarc.who.int)

This content is neither created nor endorsed by Google.

© 2024 Simba H et al. *JAMA Network Open*.

Le langage de la communication encancérologie en Afrique

Cher participant, chèreparticipante,
D’abord, nousvous remercions de prendre le temps de nous lire et d’accepter de participer à cetteenquête sur le langage de la communication sur le cancer en Afrique. Cetteenquête est menée par le Centre international de Recherche sur le Cancer del’OMS (CIRC-OMS) en collaboration avec l’Organisation Africaine pour larecherché et la formation en Cancérologie (AORTIC). Vous trouverez ci-dessousun questionnaire en ligne simple à remplir, après une courte introduction surnotre projet de recherche.
Aujourd’hui, le cancerest une des principales causes de mortalité dans le monde. Sur le continent africain,on estime que le nombre de nouveaux cas de cancer par an augmentera de 70%entre 2012 et 2030 en raison uniquement du changement démographique, ce qui provoqueune inquiétude croissante sur ce continent. En raison de cette augmentation, unepriorité majeure est actuellement accordée à la réduction de la charge ducancer dans cette région du monde en multipliant les programmes de surveillanceet de lutte contre le cancer, ainsi qu’en y ajoutant la mise en place des étudesépidémiologiques pour comprendre l'étiologie des cancers les plus fréquemmentobservés par les cliniciens.
Par ailleurs, la diversité linguistique et les difficultésliées à l'incapacité des professionnels de santé à communiquer efficacementavec les patients dans les très nombreuses langues parlées en Afrique posentproblème. En effet, la confiance entre les patients et les professionnels de santéest largement influencée par la langue de communication utilisée. La qualité dulangage a la capacité de faire adhérer un patient ou, à l’inverse, de l’empêcherà participer correctement aux soins. Le langage reste aujourd’hui un levieressentiel dans l’adhésion aux soins mais aussi dans la lutte contre le cancer.Pourtant, il a été largement sous-étudié et négligé en cancérologie dans le contexteafricain, bien que le contient africain regroupe à lui seul environ un tiers detoutes les langues parlées au monde. Il est donc crucial de bien comprendre lestermes médicaux utilisés en cancérologie dans les différentes languesafricaines et comment ces termes sont transmis par les professionnels auxpatients et à la communauté en général.
Il a donc étédécidé de conduire une enquête intitulée « Le langage de la communicationutilisée en cancérologie en Afrique ». L’objectif primordial de ce projet derecherche est 1) de collecter des informations sur les termes médicaux utilisésen cancérologie dans les langages africaines, 2) d’analyser la manière dont lelangage médical est transmis aux patients atteints du cancer et à la communautédans ces mêmes langues et 3) de mieux comprendre la signification des termes decommunication utilisés. Plus précisément, cette enquête a pour objectifs de

© 2024 Simba H et al. *JAMA Network Open*.

mettre en lumière l'existence ou l'inexistence de termes médicaux liés au cancerdans les différentes langues africaines existantes mais aussi de mieuxconnaitre la nature de la terminologie du cancer dans ces mêmes langues et lamanière dont ces termes peuvent contribuer à susciter la crainte, à engendrerdes disparités de compréhension entre les patients et à poser des difficultésde communication pour les professionnels de la santé.
Dans cetteenquête, il vous sera demandé de traduire certains termes liés au cancer — plusparticulièrement ceux utilisés dans le diagnostic et le traitement — dans leslangues africaines que vous parlez et puis de les retraduire directement en français.
Nous invitonstous les professionnels de santé, les agents de santé communautaire, leschercheurs ainsi que les scientifiques impliqués dans les soins du cancer àparticiper à cette enquête. Nous espérons couvrir les termes liés au cancer danstous les pays d'Afrique et toutes les langues principales de chaque pays. Nous vousinvitons donc à participer à cette enquête mais aussi à partager le lien de l'enquêteavec d'autres professionnels concernés et en particulier ceux d'autre pays d'Afrique.
Les informations recueilliespermettront de mettre en lumière l'état de la communication sur le cancer enAfrique, ce qui pourrait, à terme, servir à mettre en place une plateforme qui seraune référence pour les travaux futurs sur le sujet. Les données collectéesseront analysées et publiées sous forme d'un article scientifique qui serasoumis à un comité de lecture. Par ailleurs, vous êtes également invités àfaire partie l'équipe en fournissant vos coordonnées à la fin de l'enquête.
Vous pouvez répondre à cette enquête une fois pour chaque langue que vousparlez.
Nous vousremercions par avance du temps que vous consacrerez à notre enquête et detoutes les informations fournies. Si vous souhaitez fournir ou obtenir desinformations supplémentaires, veuillez envoyer un courriel à :
Dr. Hannah Simba Chercheurepostdoctoral, Branche Epidémiologie de l'environnement et du mode de vie (ENV)Centre international de recherche sur le cancer (CIRC) de l'organisationmondial de la santé (OMS)150 cours Albert Thomas69372 Lyon CEDEX 08, FranceCourriel : simbah@iarc.who.int.https://www.iarc.who.int/staff_member/hannah-simba/
Au nom du Dr. MiriamMutebi, Dr. Moses Galukande, Dr Adamu Addissie, Dr Efua Prah, and Dr. Valerie
McCormack(CIRC-OMS)
* Indicates required question

Formulaire de consentement destiné aux participant(e)s de l'enquête

L'objectif denotre projet de recherche est de recueillir des informations sur la manièredont les termes liés au cancer sont communiqués par les professionnels auxpatients et à la communauté en général dans les langues africaines. Ce projet derecherche est mené par le Centre international de recherche sur le cancer(CIRC-OMS) en collaboration avec l'Organisation africaine pour la recherche etla formation sur le cancer (AORTIC). Vous êtes invité(e)s à participer à ceprojet de recherche si vous êtes un professionnel de santé exerçant dans ledomaine de la cancérologie, un agent de santé communautaire ou si vous êtes unchercheur scientifique dans le domaine du cancer.
Votreparticipation à ce projet de recherche est volontaire, non rétribuée mais nécessite la signature du consentement électroniqueci-dessous. Cela signifie que vous êtes donc libred'accepter ou de refuser d'y participer. Vous pouvez également vous retirer dece projet à n'importe quel moment, sans avoir à en donner les raisons, enfaisant connaître votre décision à l'investigateur principal de ce projet. Nousrappelons que votre décision de ne pas participer à ce projet de recherche oude vous en retirer n'implique aucune pénalité.
La procédureconsiste à remplir un questionnaire en ligne simple qui vous prendra moins de 30min de votre temps. Vos réponses resteront strictement confidentielles. Parailleurs, nous ne recueillerons aucune information permettant de vousidentifier, telles que votre nom, votre

© 2024 Simba H et al. *JAMA Network Open*.

adresse électronique et votre adresse IP.Les questions du formulaire porteront sur la traduction en langues africaines destermes les plus fréquemment utilisés dans le diagnostic et le traitement ducancer.
Lorsque vousparticipez à ce projet de recherche, soyez assuré(e) que nous ferons tout pourprotéger la confidentialité de vos renseignements. Toutes les données sontstockées dans un format électronique et sont protégées par un mot de passeempêchant leur identification En revanche, votre accord à participer impliqueque vous acceptez que le responsable du projet puisse utiliser les informationscollectées à des fins scientifiques.
Pour toutequestion ou demande sur ce projet de recherche, vous pouvez contacter le Dr.Hannah Simba au simbah@iarc.who.int.

Mercide compléter les informations ci-dessous et de cocher la case si vous consentezà ce qui précède
1.

1. **CONSENTEMENT ELECTRONIQUE :**
Veuillez sélectionner parmi les choix ci-dessous. En cliquant sur le bouton« accepter » ci-dessous, vous indiquez que :
a. Vous avez lu et accepté les informations ci-dessous
b. Vous décidez de participer volontairement à ce projet et que vous neserez pas rémunéré(e)s.
c. Vous avez au moins 18 ans
Si vous ne souhaitez pas participer à cette enquête, veuillez refuser departiciper en cliquant sur le bouton "ne pas accepter"
*
Check all that apply.
Accepter

Ne pas accepter

Données démographiques

2.

Nous vous demandons de remplir un formulaire pour chaque langue du paysdans lequel l’expérience a lieu. De quel pays provient votre expérience ?
Mark only one oval.
Afrique du Sud
Algérie
Angola
Bénin
Botswana
Burkina Faso
Burundi
Cameroun
Cap-Vert
République centrafricaine

© 2024 Simba H et al. *JAMA Network Open*.

Comores
République du Congo
République démocratique du Congo
Côte d'Ivoire
Djibouti
Égypte
Érythrée
Eswatini
Éthiopie
Gabon
Gambie
Ghana
Guinée
Guinée-Bissau
Guinée équatoriale
Kenya
Lesotho
Liberia
Libye
Madagascar
Malawi

Mali
Maroc
Maurice
Mauritanie
Mozambique
Namibie
Niger
Nigeria
Ouganda
Rwanda
São Tomé-et-Principe
Sénégal
Seychelles
Sierra Leone
Somalie
Soudan
Soudan du Sud
Tanzanie
Tchad
Togo
Tunisie
Zambie
Zimbabwe
Option 55

3.

Dans quelle tranche d'âge vous situez-vous ?
*
Mark only one oval.

© 2024 Simba H et al. *JAMA Network Open*.

18-40
41-60
60+

4.

Quelle est votre identité de genre?
Veuillez cocher toutes les cases qui s'appliquent
*
Check all that apply.
Femme

Homme

Non-binaire/non-conforme

Transgenre

Je préfère ne pas le dire

Autre

Other:

5.

Veuillez cocher la case correspondant à votre réponse
*
Mark only one oval.
Oncologue
Infirmière en oncologie
Agent de santé communautaire
Chercheur en cancérologie
Travailleur dans le domaine de la santé
Guérisseurs traditionnels
Autre

Other:
6.

Quel est le nom de votre langue (écrire le nom de la langue) ?
*
7.

Le cas échéant, à quel groupe ethnique votre langue appartient-elle (écrire lenom du groupe ethnique) ?
Traduction et explication des termes utilisés en cancérologie dans votrelangue

© 2024 Simba H et al. *JAMA Network Open*.

Dans cette section, nous vous demanderons detraduire certains termes et mots utilisés pour désigner le cancer dans votrepropre langue, puis donner la définition en français (si une traductionexiste). Veuillez utiliser l'espace prévu pour toute explication ou commentairesupplémentaire concernant le terme traduit et sa signification. Si vous neconnaissez pas la traduction ou si un terme n'existe pas dans votre langue, vouspouvez laisser la réponse en blanc.

8.

Comment appelez-vous le terme "cancer" dans votre langue ? (écrire le nomdans votre langue)

9.

Quelle est la traduction la plus proche de ce terme en français ? (écrire latraduction en français)*Veuillez utiliser l'espace prévu pour donner toute information supplémentaire quevous souhaiteriez nous communiquer sur la signification de ce terme*

10.

Comment appelez-vous le terme "tumeur" dans votre langue ? (écrire le termedans votre langue)

11.

Quelle est la traduction la plus proche de ce terme en français ? (écrire latraduction en français)

Veuillez utiliser l'espace prévu pour donner toute information supplémentaireque vous souhaiteriez nous communiquer sur la signification de ce terme.

12.

Comment appelez-vous le terme "bénigne" dans votre langue ? (écrire le nomdans votre langue)

13.

Quelle est la traduction la plus proche de ce terme en français ? (écrire latraduction en français)

Veuillez utiliser l'espace prévu pour donner toute information supplémentaireque vous souhaiteriez nous communiquer sur la signification de ce terme.

14.

Comment appelez-vous le terme "maligne" dans votre langue ? (écrire le nomdans votre langue)

© 2024 Simba H et al. *JAMA Network Open*.

15.

Quelle est la traduction la plus proche de ce terme en français ? (écrire latraduction en français)

Veuillez utiliser l’espace prévu pour donner toute information supplémentaireque vous souhaiteriez nous communiquer sur la signification de ce terme.

16.

Comment appelez-vous le terme "Biopsie" dans votre langue ? (écrire le termedans votre langue)

17.

Quelle est la traduction la plus proche de ce terme en français ? (écrire latraduction en français)

Veuillez utiliser l’espace prévu pour donner toute information supplémentaireque vous souhaiteriez nous communiquer sur la signification de ce terme.

18.

Comment appelez-vous le terme " chronique" dans votre langue ? (écrire leterme dans votre langue)

19.

Quelle est la traduction la plus proche de ce terme en français ? (écrire latraduction en français)

Veuillez utiliser l’espace prévu pour donner toute information supplémentaireque vous souhaiteriez nous communiquer sur la signification de ce terme.

20.

Comment appelez-vous le terme "Métastase" dans votre langue ? (écrire leterme dans votre langue)

21.

Quelle est la traduction la plus proche de ce terme en français ? (écrire latraduction en français)

Veuillez utiliser l’espace prévu pour donner toute information supplémentaireque vous souhaiteriez nous communiquer sur la signification de ce terme.

22.

Comment appelez-vous le terme "chimiothérapie" dans votre langue ? (écrire leterme dans votre langue)

23.

© 2024 Simba H et al. *JAMA Network Open*.

Quelle est la traduction la plus proche de ce terme en français ? (écrire latraduction en français)
Veuillez utiliser l'espace prévu pour donner toute information supplémentaireque vous souhaiteriez nous communiquer sur la signification de ce terme.
24.

Comment appelez-vous le terme "Radiation ou Radiothérapie" dans votrelangue ? (écrire le terme dans votre langue)
25.

Quelle est la traduction la plus proche de ce terme en français ? (écrire latraduction en français)
Veuillez utiliser l'espace prévu pour donner toute information supplémentaireque vous souhaiteriez nous communiquer sur la signification de ce terme.
26.

Comment appelez-vous le terme "stade du cancer " dans votre langue ? (écrirele terme dans votre langue)

27.

Quelle est la traduction la plus proche de ce terme en français ? (écrire latraduction en français)
Veuillez utiliser l'espace prévu pour donner toute information supplémentaireque vous souhaiteriez nous communiquer sur la signification de ce terme.
28.

Comment appelez-vous le terme "Chirurgie" dans votre langue ? (écrire le termedans votre langue)
29.

Quelle est la traduction la plus proche de ce terme en français ? (écrire latraduction en français)
Veuillez utiliser l'espace prévu pour donner toute information supplémentaireque vous souhaiteriez nous communiquer sur la signification de ce terme.
30.

Comment appelez-vous le terme "essai clinique " dans votre langue ? (écrire leterme dans votre langue)

31.

Quelle est la traduction la plus proche de ce terme en français ? (écrire latraduction en français)
Veuillez utiliser l'espace prévu pour donner toute information supplémentaireque vous souhaiteriez nous communiquer sur la signification de ce terme.

32.

Comment appelez-vous le terme "Palliation ou les soins palliatifs" dans votrelangue ? (écrire le terme dans votre langue)
33.

Quelle est la traduction la plus proche de ce terme en français ? (écrire latraduction en français)
Veuillez utiliser l'espace prévu pour donner toute information supplémentaireque vous souhaiteriez nous communiquer sur la signification de ce terme.
34.

Comment appelez-vous le terme "récidive" dans votre langue ? (écrire le termedans votre langue)

35.

Quelle est la traduction la plus proche de ce terme en français ? (écrire latraduction en français)
Veuillez utiliser l'espace prévu pour donner toute information supplémentaireque vous souhaiteriez nous communiquer sur la signification de ce terme.
36.

Comment appelez-vous le terme "survie" dans votre langue ? (écrire le termedans votre langue)
37.

Quelle est la traduction la plus proche de ce terme en français ? (écrire latraduction en français)
Veuillez utiliser l'espace prévu pour donner toute information supplémentaireque vous souhaiteriez nous communiquer sur la signification de ce terme.
38.

Comment appelez-vous le terme "rémission" dans votre langue ? (écrire leterme dans votre langue)

39.

Quelle est la traduction la plus proche de ce terme en français ? (écrire latraduction en français)
Veuillez utiliser l'espace prévu pour donner toute information supplémentaireque vous souhaiteriez nous communiquer sur la signification de ce terme.

Traduction de différents types de cancer

© 2024 Simba H et al. *JAMA Network Open*.

Danscette section, nous vous demanderons de fournir les noms de différents types decancer qui existent dans votre langue
40.

S'il existe de différents types de cancer (sites) qui ont des traductionsspécifiques ou difficiles dans votre langue, veuillez les énumérer ici enrespectant le format suivant : nom du cancer en français, nom du cancer dansvotre langue
41.

Avez-vous d’autres idées ou commentaires à nous faire sur ce sujet ?

Remerciement et coordonnées
Nous vous remercions une fois de plus d’avoir pris le temps de répondre à cette enquête. N’hésitez pas à nous contacter si vous souhaitez vous impliquer d'avantage ou faire partie de l’équipe organisatrice du projet au mail suivant : simbah@iarc.who.int
Mille mercis.
Il s’agit d’un projet scientifique. Le contenu du projet n’est ni approuvé ni parrainé par Google.

This content is neither created nor endorsed by Google.

© 2024 Simba H et al. *JAMA Network Open*.

A linguagem da comunicação sobre ocancro na África

CaroParticipante,
Obrigado por tomaro seu tempo para ler e participar desta pesquisa. Faremos aqui uma breveintrodução e a pesquisa se segue.
Na AgênciaInternacional para Pesquisa sobre o Cancro (IARC/WHO) e em colaboração com aOrganização Africana de Pesquisa e Formação sobre o Cancro (AORTIC), estamos arealizar uma pesquisa sobre "A Linguagem da Comunicação sobre o Cancro naÁfrica". O objetivo é recolher informações sobre a linguagem médicarelacionada ao cancro e o seu tratamento: como comunica-se com os pacientes eas comunidades e o que significam estes termos nas línguas africanas. O estudosalientará a existência ou não de termos relacionados ao cancro, bem como anatureza da terminologia do cancro nas línguas africanas e como isso pode influenciaro medo, as disparidades entre os pacientes, além de estabelecer dificuldades decomunicação para os profissionais de saúde. O estudo recolherá informaçõessobre termos oncológicos traduzidos para as línguas africanas. Solicitaremosque você traduza termos selecionados, relacionados ao cancro, (na sua maioriaos utilizados no diagnóstico e tratamento) para a sua língua africana, e depoisos traduza diretamente de volta para inglês.
Convidamos todosos profissionais de saúde, pesquisadores e cientistas envolvidos nos cuidadosoncológicos a participar neste estudo. Esperamos incluir todos os paísesafricanos e todas as principais línguas de cada país; desse modo, pedimos-lheque compartilhe o link deste estudo com outros profissionais relevantes eespecialmente os de outros países. Acreditamos que ainformação recolhida a partir desta pesquisa irá demonstrar o estado dacomunicação sobre o cancro na África e servir de plataforma e base para futurostrabalhos sobre o tema. A informação recolhida nesta pesquisa será analisada epublicada num manuscrito. Você está convidado a fazer parte da equipe,fornecendo os seus dados de contacto no final da pesquisa.
Você poderárealizar esta pesquisa uma vez para cada língua que você fale.
Obrigado pelo seutempo e por todas as informações fornecidas. Se desejar fornecer ou obter maisinformações, envie um e-mail à Dra. Hannah Simba (simbah@iarc.who.int)
Dra. Hannah Simba,https://www.iarc.who.int/staff_member/hannah-simba/ Secção de Ambientee Epidemiologia do Estilo de Vida, Agência Internacional para pesquisa sobre oCancro
Em nome da Dra.Miriam Mutebi, Dr. Moses Galukande, Dr Adamu Addissie, Dr Efua Prah, e Dra. Valerie McCormack(IARC)
* Preenchimentoobrigatório
* Indicates required question

Termo de consentimento
O objetivo deste projetode pesquisa é recolher informações sobre como os termos relacionados ao cancrosão comunicados aos pacientes e às comunidades em línguas africanas. Este é um projetode pesquisa a ser conduzido pela Agência Internacional para Pesquisa sobre o Cancro(IARC/OMS) em colaboração com a AORTIC. Você está convidado a participar neste projetode pesquisa porque é um profissional de saúde que trabalha nos cuidados desaúde contra o cancro, ou um investigador de cancro.
A suaparticipação nesta pesquisa é voluntária. Você pode optar por não participar.Se decidir participar desta pesquisa, você pode solicitar sua saída a qualquermomento. Se

© 2024 Simba H et al. *JAMA Network Open*.

decidir não participar neste estudo ou se se retirar daparticipação em qualquer altura, você não será penalizado.
O procedimentoenvolve o preenchimento de uma pesquisa online que demorará aproximadamente 30minutos. As suas respostas serão confidenciais, e não recolheremos informaçõesde identificação como o seu nome, endereço de correio eletrónico ou endereçoIP. As perguntas da pesquisa serão sobre traduções de termos relacionados aocancro utilizados no diagnóstico e tratamento em línguas africanas.
Faremos o nossomelhor para manter a sua informação confidencial. Todos os dados sãoarmazenados num formato eletrónico protegido por palavra-chave. Para ajudar aproteger a sua confidencialidade, as pesquisas não conterão informações que oidentifiquem pessoalmente. Os resultados deste estudo serão utilizados apenaspara fins académicos.
Se tiverquaisquer perguntas sobre o estudo, contacte a Dra. Hannah Simba(simbah@iarc.who.int).

1.

CONSENTIMENTO ELECTRÓNICO: Por favor, selecione a sua escolha abaixo.Ao clicar no botão "concordo" abaixo indica que:- leu a informação acima -concorda voluntariamente em participar - tem pelo menos 18 anos de idade. Senão deseja participar no estudo, por favor recuse a participação ao clicar nobotão "discordo".
Marque todas as alternativas que se apliquem.
*
Check all that apply.
Concordo

Discordo

Demográficos

2.

Solicitamos que preencha um formulário para cada língua. De que país é a suaexperiência?
Marque apenas uma alternativa.
Mark only one oval.
Argélia
Angola
Benim
Botsuana
Burkina Faso
Burundi
Cabo Verde
Camarões
República Centro-Africana (RCA)
Chade
Comores

© 2024 Simba H et al. *JAMA Network Open*.

República Democrática do Congo
República do Congo
Costa do Marfim
Djibouti
Egipto
Guiné Equatorial
Eritreia
Eswatini
Etiópia
Gabão
Gâmbia
Gana
Guiné
Guiné-Bissau
Quénia
Lesotho
Libéria
Líbia
Madagáscar

Malawi
Mali
Mauritânia
Maurícia
Marrocos
Moçambique
Namíbia
Níger
Nigéria
Ruanda
São Tomé e Príncipe
Senegal
Seychelles
Serra Leoa
Somália
África do Sul
Sudão do Sul
Sudão
Tanzânia
Togo
Tunísia
Uganda
Zâmbia
Zimbabué
3.

Qual é a sua idade?
Marcar apenas uma alternativa.
*
Mark only one oval.
18-40

© 2024 Simba H et al. *JAMA Network Open*.

41-60
60+

4.

Qual é a sua identidade de género?
Marque todas as alternativas que se apliquem
*
Check all that apply.
Mulher

Homem

Não-binário/não-conforme

Transgénero

Prefiro não dizer

Outros:

Other:

5.

Quem é você?
*
Mark only one oval.
Oncologista
Enfermeira de Oncologia
Trabalhador Comunitário de Saúde
Pesquisador de cancro
Trabalhador dos cuidados de saúde
Curandeiro tradicional
6.

Qual é o nome da sua língua?
*
7.

Se aplicável, a que grupo étnico pertence a sua língua?

Tradução e explicação de termos relacionados ao cancro
Nesta secção,pedimos-lhe que traduza termos selecionados, relacionados ao cancro, para a suaprópria língua e depois os traduza diretamente para inglês (se existir umatradução). Por favor, utilize o espaço fornecido para quaisquer explicações oucomentários adicionais sobre o termo traduzido e o seu significado. Se nãosouber a tradução ou se um termo não existir na sua língua, pode deixar aresposta em branco
8.

Qual é o nome de "cancro" na sua língua?
9.

What is the nearest English translation of this term?
Use the space provided to give any additional information that you would like usto know regarding the meaning of this term.
10.

Como se chama "Tumor" na sua língua?
11.

Qual é a tradução inglesa mais próxima deste termo?
Utilize o espaço fornecido para dar qualquer informação adicional que gostariaque soubéssemos sobre o significado deste termo.

12.

Qual é o nome "Benigno" na sua língua?
13.

Qual é a tradução inglesa mais próxima deste termo?
Utilize o espaço fornecido para dar qualquer informação adicional que gostariaque soubéssemos sobre o significado deste termo.
14.

Como se chama "Maligno" na sua língua?
15.

Qual é a tradução inglesa mais próxima deste termo?
Utilize o espaço fornecido para dar qualquer informação adicional que gostariaque soubéssemos sobre o significado deste termo.
16.

Como se chama "Biópsia" na sua língua?

17.

What is the nearest English translation of this term?
Use the space provided to give any additional information that you would like usto know regarding the meaning of this term.
18.

Como se chama "Crónico" na sua língua?

© 2024 Simba H et al. *JAMA Network Open*.

19.

Qual é a tradução inglesa mais próxima deste termo?
Utilize o espaço fornecido para dar qualquer informação adicional que gostariaque soubéssemos sobre o significado deste termo.
20.

Como se chama "Metástase" na sua língua?

21.

Qual é a tradução inglesa mais próxima deste termo?
Utilize o espaço fornecido para dar qualquer informação adicional que gostariaque soubéssemos sobre o significado deste termo.
22.

Qual é o nome de "Quimioterapia" na sua língua?
23.

Qual é a tradução inglesa mais próxima deste termo?
Utilize o espaço fornecido para dar qualquer informação adicional que gostariaque soubéssemos sobre o significado deste termo.
24.

Como se chama "Radiação ou Radioterapia" na sua língua?

25.

Qual é a tradução inglesa mais próxima deste termo?
Utilize o espaço fornecido para dar qualquer informação adicional que gostariaque soubéssemos sobre o significado deste termo.
26.

Como se chama "estadiamento do cancro" na sua língua?
27.

Qual é a tradução inglesa mais próxima deste termo?
Utilize o espaço fornecido para dar qualquer informação adicional que gostariaque soubéssemos sobre o significado deste termo.
28.

Qual é o nome de "Cirurgia" na sua língua?

© 2024 Simba H et al. *JAMA Network Open*.

29.

Qual é a tradução inglesa mais próxima deste termo?
Utilize o espaço fornecido para dar qualquer informação adicional que gostariaque soubéssemos sobre o significado deste termo.
30.

Como se chama "ensaio clínico" na sua língua?
31.

Qual é a tradução inglesa mais próxima deste termo?
Utilize o espaço fornecido para dar qualquer informação adicional que gostariaque soubéssemos sobre o significado deste termo.
32.

Como se chama "Paliação ou Cuidados Paliativos" na sua língua?

33.

Qual é a tradução inglesa mais próxima deste termo?
Utilize o espaço fornecido para dar qualquer informação adicional que gostariaque soubéssemos sobre o significado deste termo.
34.

Como se chama " Recorrência " na sua língua?
35.

Qual é a tradução inglesa mais próxima deste termo?
Utilize o espaço fornecido para dar qualquer informação adicional que gostariaque soubéssemos sobre o significado deste termo.
36.

Como se chama "Sobrevivência" na sua língua?

37.

Qual é a tradução inglesa mais próxima deste termo?
Utilize o espaço fornecido para dar qualquer informação adicional que gostariaque soubéssemos sobre o significado deste termo.
38.

Como se chama "Remissão" na sua língua?
39.

© 2024 Simba H et al. *JAMA Network Open*.

Qual é a tradução inglesa mais próxima deste termo?
Utilize o espaço fornecido para dar qualquer informação adicional que gostariaque soubéssemos sobre o significado deste termo.

Tradução de tipos de cancro

Nesta secçãoiremos pedir-lhe que nos indique os nomes dos tipos de cancro que existem na sualíngua

40.

Se houver tipos de cancro (sítios) que tenham traduções específicas, interessantes, ou desafiantes na sua língua, liste-os aqui no seguinte formato: nome do cancro em Inglês – nome do cancro na sua língua
41.

Tem alguma reflexão adicional, comentários sobre este tópico?

Obrigado e contacto

Obrigado por ter dedicado o seu tempo para completar esta pesquisa. Se desejar participar mais neste estudo, envie-nos por favor um e-mail. Favor contactar Dr Hannah Simba (simbah@iarc.who.int)
Este conteúdo não é criado nem endossado pelo Google.

This content is neither created nor endorsed by Google.

© 2024 Simba H et al. *JAMA Network Open*.

لغة التواصل مع مرضى السرطان في أفريقيا

عزيزي المشارك،

نشكرك على الوقت الذي استغرقته في قراءة هذا الاستطلاع والمشاركة فيه.

نحيطك علما بأن هنا توجد مقدمة موجزة ويتبعها الاستبيان (انظر الأسئلة أسفله).

وبالتعاون مع المنظمة الأفريقية للبحث والتدريب في (IARC/WHO) في الوكالة الدولية لأبحاث السرطان مجال السرطان (AORTIC)، نقوم بإجراء دراسة استقصائية حول "لغة التواصل مع مرضى السرطان في أفريقيا".

الهدف هنا هو جمع المعلومات حول كيفية التواصل مع المرضى عبر المصطلحات الطبية للسرطان وعلاجه وكذلك تفسير ما تعنيه هذه المصطلحات حسب لغات كل المجتمعات الأفريقية.

ستسلط الدراسة الضوء على وجود أو عدم وجود مفردات السرطان في اللغات الأفريقية وطبيعتها من أجل تحديد كيف يمكن أن تساهم في الخوف والتفاوتات للمرضى وتشكيل صعوبات في التواصل مع مهنيي الصحة.

وستجمع الدراسة الاستقصائية معلومات عن مصطلحات السرطان المترجمة إلى اللغات الأفريقية. سيطلب منك أن تترجم مصطلحات السرطان المقترحة (معظمها تستخدم في التشخيص والعلاج) إلى لغتك الأفريقية وأن تترجمها مباشرة إلى اللغة الانجليزية.

ندعو جميع مهنيي الصحة والعلماء والباحثين بمجال رعاية مرضى السرطان للمشاركة في هذه الدراسة.

نأمل أن نستطيع الحصول على انخراط جميع البلدان الأفريقية وبالتالي جميع اللغات الرئيسية لكل بلد، ولهذا ندعوك لمشاركة رابط الاستطلاع مع جميع مهني الصحة ذوي الصلة خاصة الذين ينتمون للبلدان الأخرى.

نتصور أن المعلومات التي سيتم جمعها من هذا الاستطلاع ستسلط الضوء على حالة التواصل بخصوص مرض السرطان في أفريقيا، وأن تكون بمثابة منصة وركيزة لأساسيات العمل المستقبلي فيما يتعلق بهذا الموضوع.

سيتم تحليل المعلومات التي تم جمعها في هذا الاستطلاع ونشرها في مخطوطة.

يمكنك إجراء هذا الاستطلاع مرة واحدة لكل لغة تتحدث بها.

شكرا لك على وقتك وعلى جميع المعلومات التي قدمتها.

إذا كنت ترغب في تقديم او الحصول على المزيد من المعلومات، يرجى إرسال بريد إلكتروني إلى الدكتورة هانا سيمبا (simbah@iarc.who.int)

Dr Hannah Simba, https://www.iarc.who.int/staff_member/hannah-simba/

فرع البيئة ونمط الحياة، علم الاوبئة، الوكالة الدولية لبحوث السرطان.

نيابة عن:

Dr Miriam Mutebi, Dr Moses Galukande, Adam Addissie, Dr Valerie McCormack (IARC)

© 2024 Simba H et al. *JAMA Network Open*.

المطلوب*:

نموذج الموافقة:

الغرض من هذا المشروع البحثي هو جمع المعلومات حول كيفية توصيل مصطلحات السرطان للمرضى وفي المجتمع باللغات الأفريقية.

بالتعاون مع (IARC/WHO) نعلمكم بأن هذا المشروع بحثي، تجربه الوكالة الدولية لأبحاث السرطان AORTIC. أنت مدعو للمشاركة في هذا المشروع البحثي لكونك مهني الصحة وتعمل بمجال رعاية السرطان، أو أنك باحث في مجال السرطان.

نعلمك أيضا ان مشاركتك في هذه الدراسة البحثية تطوعية. يمكنك اختيار عدم المشاركة. إذا قررت المشاركة في هذا الاستطلاع البحثي، فيمكنك الانسحاب في أي وقت.

إذا قررت عدم المشاركة في هذه الدراسة أو إذا انسحبت من المشاركة في أي وقت، فلن تتم معاقبتك.

يتضمن الإجراء ملء استبيان عبر الإنترنت الذي يستغرق حوالي 30 دقيقة.

نعلمك أن أجوبتك ستكون سرية، كما أننا لا نجمع معلومات تعريفية مثل الاسم أو عنوان البريد الإلكتروني أو عنوان IP، بل ستجد أن مجموع أسئلة الاستطلاع سيكون حول ترجمات مصطلحات ومفردات السرطان المستخدمة في التشخيص والعلاج إلى اللغات الأفريقية.

سنقوم بالعمل جاهدا على سرية معلوماتك. وسيتم تخزين جميع البيانات بتنسيق إلكتروني محمي بكلمة مرور لضمان سريتك كما ان الاستطلاعات لن تشمل المعلومات التي من شأنها أن تكشف هويتك الشخصية. وسيتم استخدام نتائج هذه الدراسة لأغراض علمية فقط.

إذا كانت لديك أي أسئلة او استفسارات حول الدراسة البحثية، يرجى الاتصال بالدكتورة هانا سيمبا.

(simbah@iarc.who.int)

1. الموافقة الإلكترونية: يرجى تحديد اختيارك أسفله.

يشير الضغط فوق الزر "موافق" أسفله إلى:

- لقد قرأت المعلومات أعلاه
- توافق على التطوع للمشاركة
- يبلغ عمرك 18 عامًا على الأقل

إذا كنت لا ترغب في المشاركة في الدراسة البحثية، فيرجى رفض المشاركة بالضغط فوق الزر "غير موافق".

© 2024 Simba H et al. *JAMA Network Open*.

تحقق من كل ما ينطبق.

موافق

غير موافق

التركيبة السكانية

سيُطلب منك إكمال نموذج واحد لكل لغة. من أي بلد هي تجربتك؟2.

ضع علامة واحدة فقط.

الجزائر

أنغولا

بنين

بوتسوانا

بوركينا فاسو

بوروندي

الرأس الأخضر

الكاميرون

جمهورية أفريقيا الوسطى

تشاد

جزر القمر

جمهورية الكونغو الديمقراطية

جمهورية الكونغو

ساحل العاج

جيبوتي

مصر

غينيا الاستوائية

إريتريا

اسواتيني

أثيوبيا

غابون

© 2024 Simba H et al. *JAMA Network Open*.

غامبيا

غانا

غينيا

غينيا بيساو

كينيا

ليسوتو

ليبيريا

ليبيا

مدغشقر

ملاوي

مالي

موريتانيا

موريشيوس

المغرب

موزمبيق

ناميبيا

النيجر

نيجيريا

رواندا

ساو تومي وبرينسيب

السنغال

سيشل

سيراليون

الصومال

جنوب أفريقيا

جنوب السودان

السودان

تنزانيا

توجو

تونس

© 2024 Simba H et al. *JAMA Network Open*.

أوغندا

زامبيا

زيمبابوي

ما هو عمرك؟ 3.

ضع علامة واحدة فقط.

18-40

41-60

60+

ما هي هويتك الجنسية؟4.

اختر كل ما ينطبق

تحقق من كل ما ينطبق.

امرأة

رجل

غير ثنائي/غير متطابق

متحول جنسيا

أفضل عدم القول

آخر:

من أنت؟ 5.

ضع علامة واحدة فقط .

طبيب الأورام

ممرض الأورام

عامل صحة المجتمع

باحث في السرطان

معالج تقليدي

آخر:

© 2024 Simba H et al. *JAMA Network Open*.

6. ما هو اسم لغتك؟

7. إذا كان كذلك، ما هي المنطقة التي تنتمي إليها لغتك في بلدك؟

ترجمة وشرح مصطلحات السرطان

في هذا القسم، سوف نطلب منك ترجمة مصطلحات السرطان المختارة إلى لغتك الخاصة ثم توجيهها إلى اللغة الإنجليزية (إذا كانت الترجمة موجودة). يرجى استخدام المساحة المقدمة لأي تفسيرات أو تعليقات إضافية بشأن المصطلح المترجم ومعناه.

إذا كنت لا تعرف الترجمة أو إذا لم يتم تواجد هذا المصطلح بلغتك، فيمكنك ترك الإجابة فارغة والانتقال إلى المصطلح التالي.

8. ماذا يسمى "السرطان" بلغتك؟

9. ما هي أقرب ترجمة إنجليزية لهذا المصطلح؟

استخدم المساحة المقدمة لإعطاء أي معلومات إضافية تريد أن تعرف بشأن معنى هذا المصطلح.

10. ماذا يسمى "الورم" بلغتك؟

11. ما هي أقرب ترجمة إنجليزية لهذا المصطلح؟

استخدم المساحة المقدمة لإعطاء أي معلومات إضافية تريد أن تعرف بشأن معنى هذا المصطلح.

12. ماذا يطلق على "حميد" بلغتك؟

13. ما هي أقرب ترجمة إنجليزية لهذا المصطلح؟

استخدم المساحة المقدمة لإعطاء أي معلومات إضافية تريد أن تعرف بشأن معنى هذا المصطلح.

14. ماذا يسمى "خبيث" بلغتك؟

15. ما هي أقرب ترجمة إنجليزية لهذا المصطلح؟

© 2024 Simba H et al. *JAMA Network Open*.

استخدم المساحة المقدمة لإعطاء أي معلومات إضافية تريد أن تعرف بشأن معنى هذا المصطلح.

16. ماذا تسمى "الخزعة" بلغتك؟

17. ما هي أقرب ترجمة إنجليزية لهذا المصطلح؟

استخدم المساحة المقدمة لإعطاء أي معلومات إضافية تريد أن تعرف بشأن معنى هذا المصطلح.

18. ماذا يسمى "مزمن" بلغتك؟

19. ما هي أقرب ترجمة إنجليزية لهذا المصطلح؟

استخدم المساحة المقدمة لإعطاء أي معلومات إضافية تريد أن تعرف بشأن معنى هذا المصطلح.

20. ماذا يسمى "الانبثاث/هجرة الخلايا السرطانية" بلغتك؟

21. ما هي أقرب ترجمة إنجليزية لهذا المصطلح؟

استخدم المساحة المقدمة لإعطاء أي معلومات إضافية تريد أن تعرف بشأن معنى هذا المصطلح.

22. ماذا يسمى "العلاج الكيميائي" بلغتك؟

23. ما هي أقرب ترجمة إنجليزية لهذا المصطلح؟

استخدم المساحة المقدمة لإعطاء أي معلومات إضافية تريد أن تعرف بشأن معنى هذا المصطلح.

24. ماذا يسمى "الإشعاع أو العلاج الإشعاعي" بلغتك؟

25. ما هي أقرب ترجمة إنجليزية لهذا المصطلح؟

استخدم المساحة المقدمة لإعطاء أي معلومات إضافية تريد أن تعرف بشأن معنى هذا المصطلح.

26. ماذا يسمى "انطلاق السرطان" بلغتك؟

© 2024 Simba H et al. *JAMA Network Open*.

27. ما هي أقرب ترجمة إنجليزية لهذا المصطلح؟

استخدم المساحة المقدمة لإعطاء أي معلومات إضافية تريد أن تعرف بشأن معنى هذا المصطلح.

28. ماذا تسمى "الجراحة" بلغتك؟

29. ما هي أقرب ترجمة إنجليزية لهذا المصطلح؟

استخدم المساحة المقدمة لإعطاء أي معلومات إضافية تريد أن تعرف بشأن معنى هذا المصطلح.

30. ماذا تسمى "التجربة السريرية" بلغتك؟

31. ما هي أقرب ترجمة إنجليزية لهذا المصطلح؟

استخدم المساحة المقدمة لإعطاء أي معلومات إضافية تريد أن تعرف بشأن معنى هذا المصطلح.

32. ماذا يسمى "التلطيف أو الرعاية الملطفة" بلغتك؟

33. ما هي أقرب ترجمة إنجليزية لهذا المصطلح؟

استخدم المساحة المقدمة لإعطاء أي معلومات إضافية تريد أن تعرف بشأن معنى هذا المصطلح.

34. ماذا يسمى "تكرار ظهور المرض بعد الشفاء" بلغتك؟

35. ما هي أقرب ترجمة إنجليزية لهذا المصطلح؟

استخدم المساحة المقدمة لإعطاء أي معلومات إضافية تريد أن تعرف بشأن معنى هذا المصطلح.

36. ماذا يسمى "البقاء" في لغتك؟

37. ما هي أقرب ترجمة إنجليزية لهذا المصطلح؟

استخدم المساحة المقدمة لإعطاء أي معلومات إضافية تريد أن تعرف بشأن معنى هذا المصطلح.

38. ماذا يسمى "انخفاض ظهور الاعراض" بلغتك؟

© 2024 Simba H et al. *JAMA Network Open*.

39. ما هي أقرب ترجمة إنجليزية لهذا المصطلح؟

استخدم المساحة المقدمة لإعطاء أي معلومات إضافية تريد أن تعرف بشأن معنى هذا المصطلح.

ترجمة أنواع السرطان

في هذا القسم، سوف نطلب منك تقديم أسماء لأنواع السرطان الموجودة في لغتك

40. إذا كانت هناك أنواع سرطان (مناطق) لها ترجمة محددة أو اهتمام أو صعبة بلغتك، فيرجى سردها بالتنسيق التالي: اسم السرطان باللغة الإنجليزية - اسم السرطان في لغتك

41. هل لديك أي أفكار إضافية، تعليقات بشأن هذا الموضوع؟

شكرا لك والاتصال

شكرا لك على قضاء الوقت لإكمال الاستبيان. إذا كنت ترغب في المشاركة بشكل أكبر في هذه الدراسة، فيرجى إرسال بريد إلكتروني إلينا. يرجى إرسال بريد إلكتروني إلى الدكتورة هانا سيمبا (simbah@iarc.who.int)

لا يتم إنشاء هذا المحتوى ولا معتمد من قِبل Google.

نماذج Google

© 2024 Simba H et al. *JAMA Network Open*.
