# Supplementary material for: Cancer Care Terminology in African Languages
Source: JAMA Netw Open. 2024 Aug 30;7(8):e2431128. doi: 10.1001/jamanetworkopen.2024.31128 (PMC11364996; doi:10.1001/jamanetworkopen.2024.31128)
Supplement: Supplement 3. — Data Sharing Statement [file jamanetwopen-e2431128-s003.pdf]

# Data Sharing Statement

Simba. Cancer Care Terminology in African Languages. *JAMA Netw Open*. Published August 30, 2024. doi:10.1001/jamanetworkopen.2024.31128

**Data**
**Data available:** Yes
**Data types:** Deidentified participant data
**How to access data:** The data has been submitted together with other manuscript files as a supplementary file
**When available:** With publication

**Supporting Documents**
**Document types:** None

**Additional Information**
**Who can access the data:** anyone requesting the data
**Types of analyses:** NA
**Mechanisms of data availability:** NA
